# Supplementary material for: High-resolution assessment of multidimensional cellular mechanics using label-free refractive-index traction force microscopy
Source: Commun Biol. 2024 Jan 20;7:115. doi: 10.1038/s42003-024-05788-4 (PMC10799850; doi:10.1038/s42003-024-05788-4)
Supplement: Supplementary file 2 — Supplementary Information [file 42003_2024_5788_MOESM2_ESM.pdf]

High-resolution assessment of multidimensional cellular mechanics using label-free refractive-index traction force microscopy: Supplementary Information

Moosung Lee[1,2,3†], Hyuntae Jeong[4†], Chaeyeon Lee[5†], Mahn Jae Lee[2, 6], Benedict Reve Delmo[7], Won Do Heo[5*], Jennifer H. Shin[4*], and YongKeun Park[1,2,8*]

[1] Department of Physics, Korea Advanced Institute of Science and Technology (KAIST), Daejeon 34141, South Korea;

[2] KAIST Institute for Health Science and Technology, KAIST, Daejeon 34141, South Korea;

[3] Current affiliation: Institute for Functional Matter and Quantum Technologies, Universität Stuttgart, 70569 Stuttgart, Germany

[4] Department of Mechanical Engineering, Korea Advanced Institute of Science and Technology (KAIST), Daejeon 34141, South Korea;

[5] Department of Biological Sciences, Korea Advanced Institute of Science and Technology (KAIST), Daejeon 34141, South Korea;

[6] Graduate School of Medical Science and Engineering, Korea Advanced Institute of Science and Technology (KAIST), Daejeon 34141, South Korea;

[7] Department of Bio and Brain Engineering, Korea Advanced Institute of Science and Technology (KAIST), Daejeon 34141, South Korea;

[8] Tomocube Inc., Daejeon 34109, South Korea

[†] These authors equally contributed to the work.

**Corresponding authors: W.D.H (wondo@kaist.ac.kr), J. S (j_shin@kaist.ac.kr) Y.K.P (yk.park@kaist.ac.kr)*

E-mail addresses

Moosung Lee: lkaaamo@gmail.com

Hyuntae Jung: jhtt2914@kaist.ac.kr

Chaeyeon Lee: chaeylee@kaist.ac.kr

Mahn Jae Lee: mjleegsmse@kaist.ac.kr

Benedict Reve Delmo: bendelmo5201@kaist.ac.kr

Won Do Heo: wondo@kaist.ac.kr

Jennifer H. Shin: j_shin@kaist.ac.kr

YongKeun Park: yk.park@kaist.ac.kr

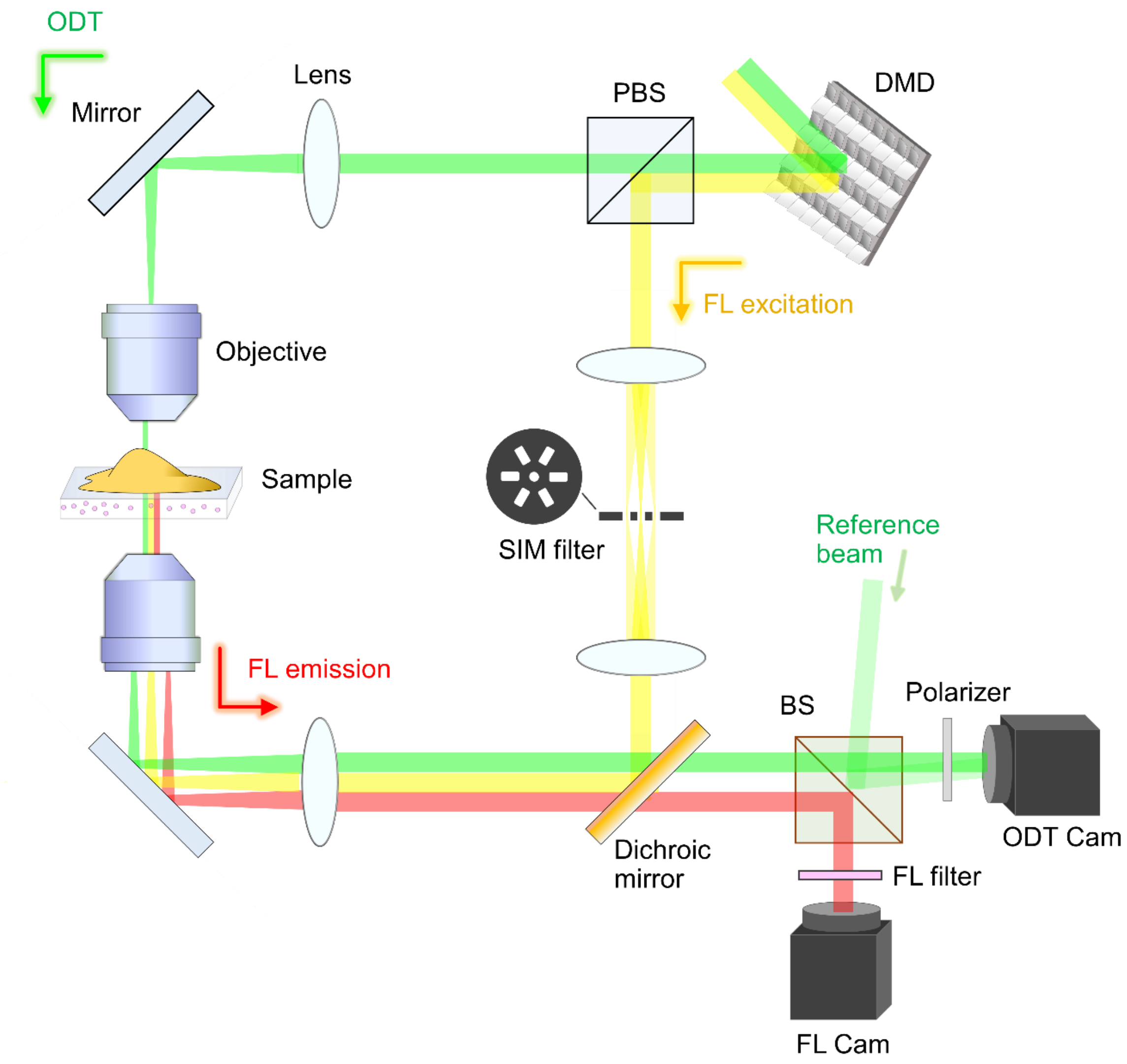

Supplementary Figure 1 | Schematic diagram of the optical setup.

DMD: digital micromirror device; PBS: polarizing beam splitter; BS: beam splitter. Green rays indicate the beam path for optical diffraction tomography (ODT). Yellow rays indicate the beam path for fluorescence excitation (FL excitation). Red rays indicate the beam path for emitted fluorescence light (FL emission).

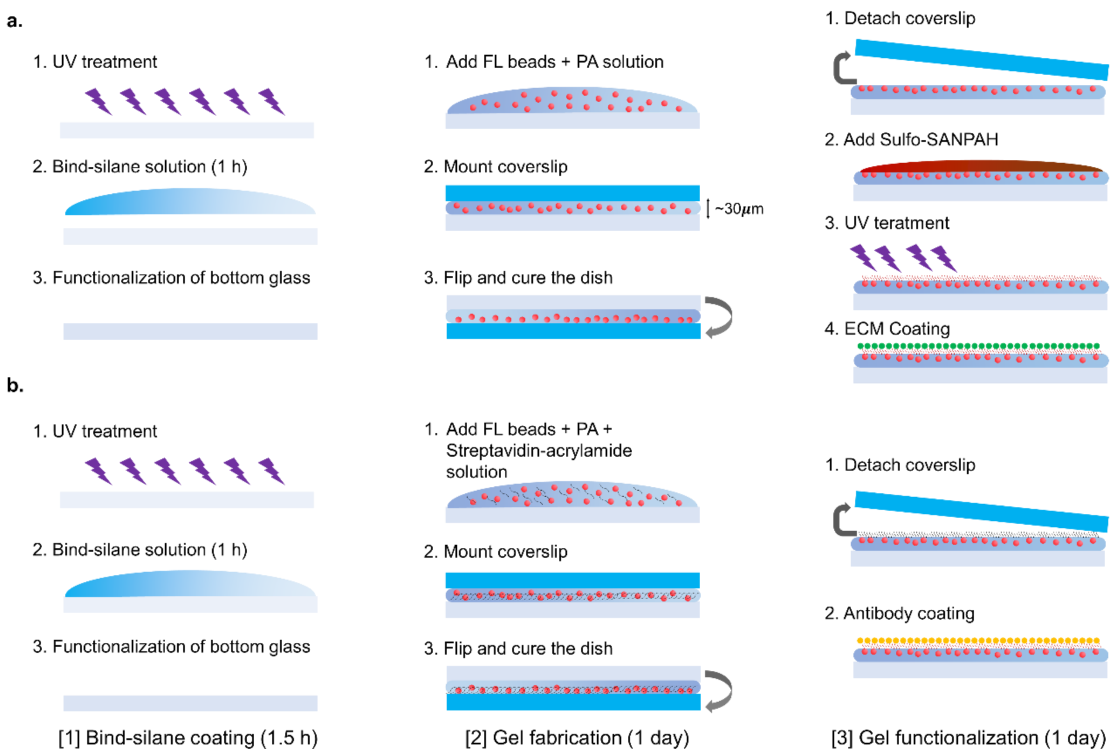

Supplementary Figure 2 | Schematic illustration of the gel fabrication.
a and b, procedure for coating **a,** extra-cellular matrix (ECM) for NIH-3T3 and MDCK cells and **b,** antibody for T cells, respectively.

Supplementary Note 1: Protocol for gel fabrication in details

To complement the experimental procedure of the gel fabrication, we provide the step-by-step procedures with details of used reagents and timing information. The schematic of the protocol is summarized in the Supplementary Fig. 2.

Used reagents: deionized water (DW); acetic acid (AA; Sigma-Aldrich, MO, USA); 3-(Trimethoxysilyl)propyl methacrylate (TMSPMA; Sigma-Aldrich, MO, USA); 2% Bis solution (Bio-Rad Laboratories Inc., CA, USA); 40% Acrylamide solution (Bio-Rad Laboratories Inc., CA, USA); ammonium persulfate (APS; Bio-Rad Laboratories Inc., CA, USA); N,N,N´,N´-Tetramethyl ethylenediamine (TEMED; Sigma-Aldrich, MO, USA); 200-nm-diameter fluorescent beads (FL beads; FluoSpheres; Life Technologies, CA, USA); streptavidin acrylamide (Invitrogen™, MA, USA); Hydroxyethyl piperazine Ethane Sulfonic acid (HEPES; Life Technologies); sulfosuccinimidyl-6-(4-azido-2-nitrophenylamino) hexanoate (Sulfo-SANPAH; Proteochem, UT, USA); Dimethyl sulfoxide

(DMSO; Sigma Aldrich); collagen type I (PureCol; Advanced BioMatrix, CA, USA); CD3 antibody (OKT3; 317326; BioLegend, CA, USA); CD28 antibody (CD28.6; # 16-0288-85; Invitrogen)

[1] Bind silane coating / Timing: 1.5 h

(1) Make bind silane solution: DW 8 mL + 3.2 μL AA + 2 μL TMSPMA. The mixed solution can be stored in long term.

(2) Expose ultraviolet (UV) lamp @ 365 nm for 5 min on the Tomodish before mounting the bind silane solution.

(3) Mount bind silane solution on Tomodish, and incubate the dish at room temperature for 1 h.

(4) Remove the solution on the dish and wash it three times with DW.

[2-1] Polyamide (PA) gel fabrication for NIH-3T3 and MDCK cell experiments / Timing: 1 day (Supplementary Fig. 2a)

(1) In a 10 mL falcon tube, prepare the PA gel solution with the following composition for the following given gel stiffnesses. Make sure that the solution is completely mixed by pipetting and vortexing.

  A. 11 kPa gel: 1781.76 μL DW + 628.56 μL 40% AA + 87.998 μL 2% Bis Solution

(2) Add 12.5 μL 10% (w/v %) APS solution.

(3) Prepare for FL beads by sonicating the original solution for 1 h. Then add 25 μL bead solution to the 250 uL gel solution.

(4) Add 1.4 μL TEMED (Sigma-Aldrich) solution. Since TEMED solution is toxic and odorous, the solution has to be prepared in a fume hood. And make sure to mix the solution thoroughly using a 1 mL pipette at most within 10 minutes. Otherwise, the substrate is not homogeneously jellified.

(5) To make a 30-μm-thick gel, add 4.1 μL solution on Tomodish and press it with a 12-mm diameter coverslip. Flip the dish, add the water to the dish, and place it in the refrigerator for a day.

[2-2] PA gel fabrication for T-cell experiments / Timing: 1 day (Supplementary Fig. 2b)

The procedure is the same as in [2-1], expect for the composition of gel solution in (1):

  A. 1.2 kPa gel for the T cell culture: 2090.7 μL DW + 245.162 μL 40% AA + 62.77 μL 2% Bis Solution + 16 μg streptavidin acrylamide.

[3-1] Coating an extracellular matrix (ECM) for adherent cells / Timing: 1 day

(1) Wash the gel with 1 mL DW. Then wash the gel again with 0.1M 1 mL HEPES solution for three times.
(2) Prepare sulfo-sampah solution.
    A. Aliquot 50 mg in 2 ml DMSO solution by 8 μl.
    B. Add 50 mM HEPES solution to the aliquoted solution until it becomes 400 uL. Caution. Since sulfo-sampah is photosensitive, prepare the sample in a clean bench with LED turned off.
    C. Mount the solution & expose UV @ 365 nm for 15 min.
    D. Aspirate solution. Wash it with HEPES solution thoroughly three times (1 min, 3 min, 5 min) until the dye is removed.
    E. Submerge the gel in DPBS solution. Expiration date: 1 month.
(3) Mount the collagen solution (50 mg/mL) & place it in the fridge for a day.

[3-2] Coating an antibody for adherent cells / Timing: 1 day

The procedure is simpler than [3-1] as the antibody coating does not require sulfo-sampah solution. Coat the gel substrate with 5 μg/mL each of anti-CD3 and anti-CD28 a day before the imaging experiment.

Calibration of the gel thickness and stiffness

Before the cell experiment, we calibrated the thickness and stiffness of the PA gel. We estimated the thickness of the gel substrate from the 3D RI/FL images of the gel embedded with the beads. We estimated the elasticity of the gels from the previous study[1].

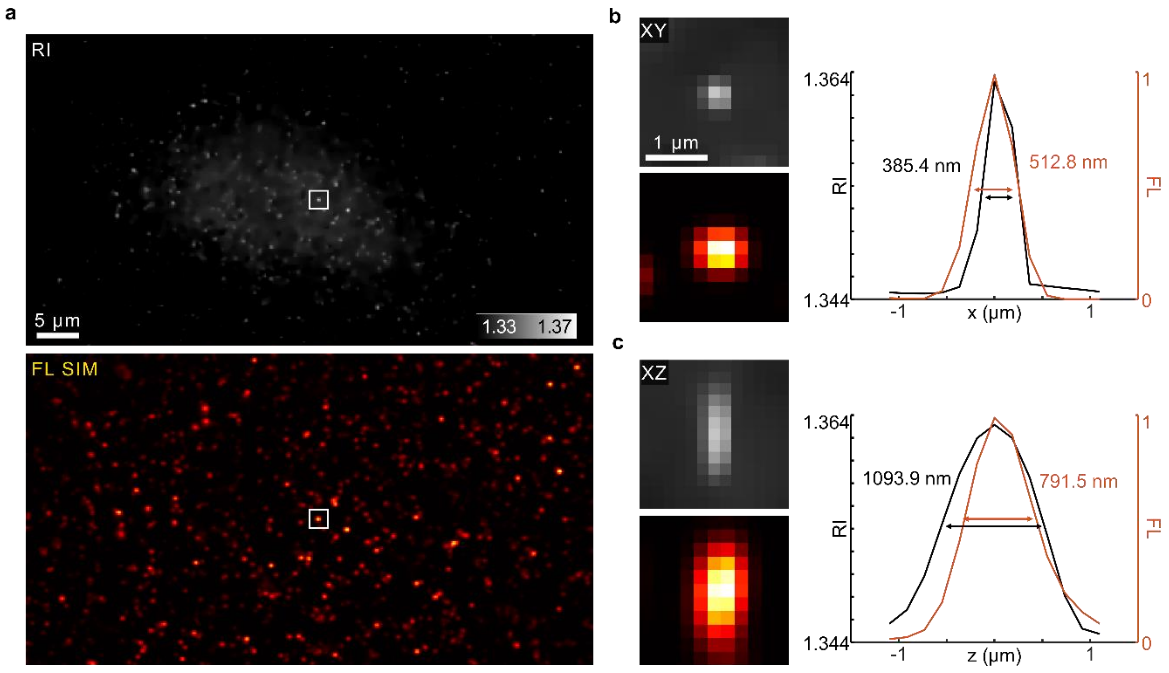

Supplementary Figure 3 | Distinctness of fiducial markers in RI-TFM.

a, 2D images of RI and FL SIM on the substrate surface. **b, c,** Magnified image of a single bead. **b,** XY cross-sections, line plots, and corresponding lateral full-width half-maximum. **c,** XZ cross-sections, line plots, and corresponding axial full-width half-maximum.

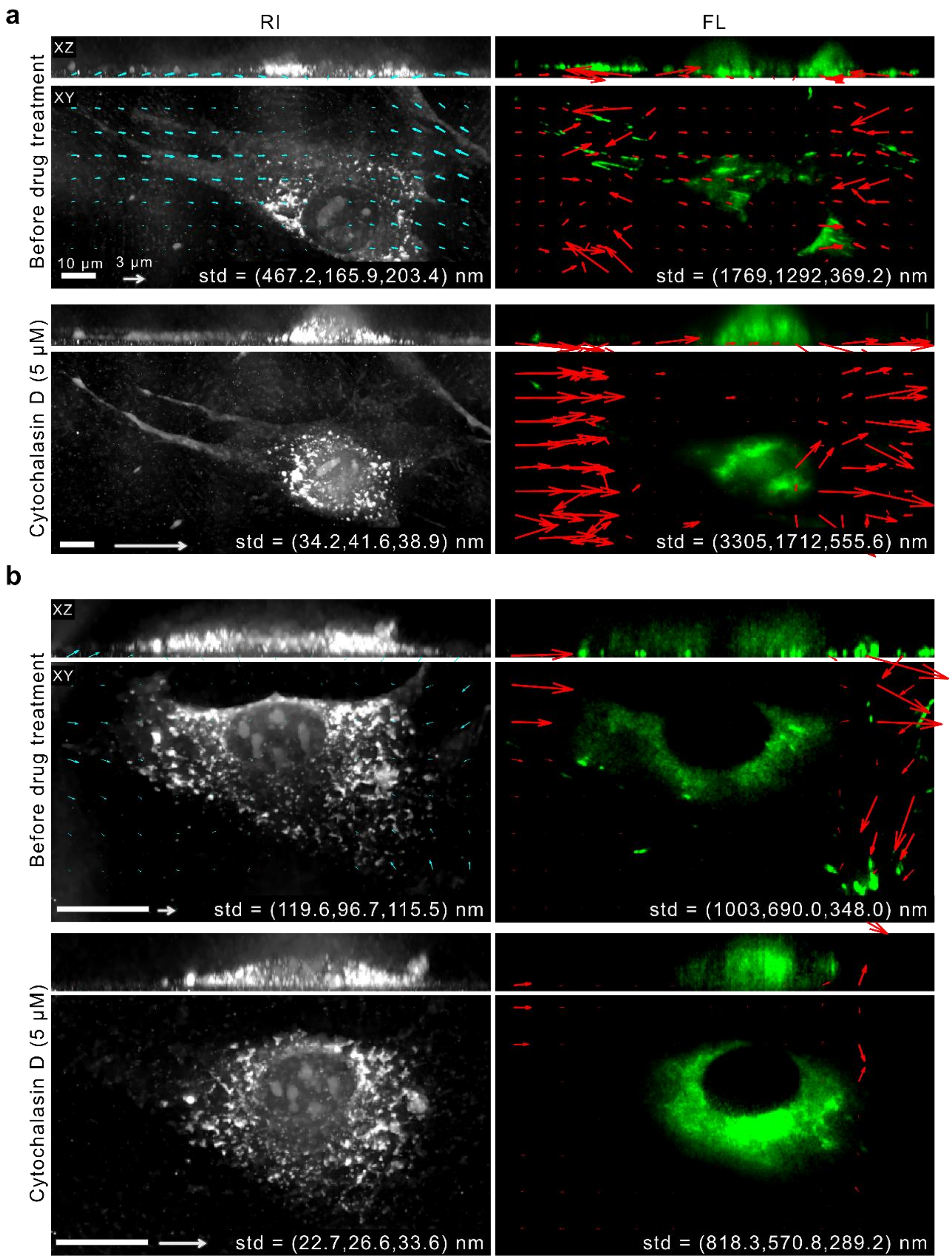

Supplementary Figure 4 | XZ and XY cross sections of refractive index (RI; grey) and FL (paxillin-EGFP; green) and 3D displacement vector for NIH3T3 cells in Fig. 3.

a, Cell 1, and **b,** Cell 2 before and 1 h after treatment with cytochalasin D (5 μM). Std indicates the standard deviations of the displacement vectors along the x, y, and z directions.

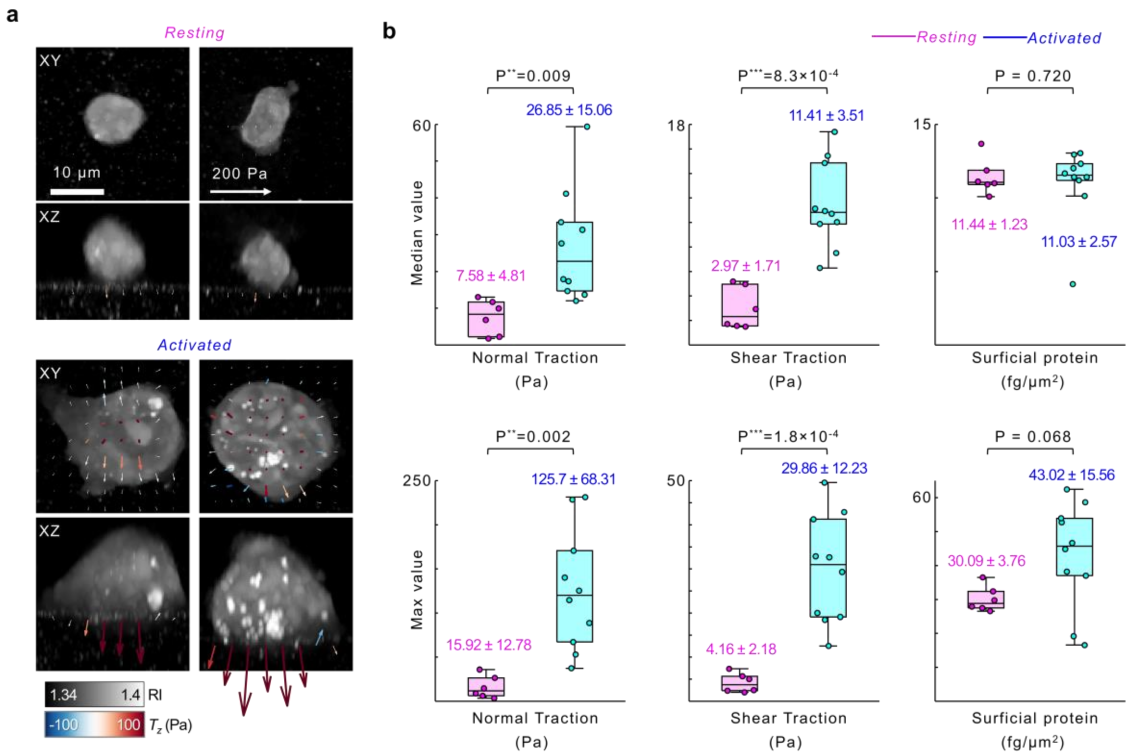

Supplementary Figure 5 | Statistical analysis of traction forces and surface protein density of human CD8+ T cells.

a, Representative RI cross-sectional images of two resting (top) and two activated (bottom) T cells. **b,** Quantification of median (top) and maximum (bottom) values for normal traction (left), shear traction (middle), and surface protein density (right). N = 6 for resting, and 10 for activated T cells. Mean ± standard deviation is shown for each boxplot. Each boxplot indicates the median, upper, lower quartiles, and 1.5x interquartile range of each population. *: $P < 0.05$, **: $P < 0.01$, ***: $P < 0.001$ for the two-sided, unpaired Student's t-test.

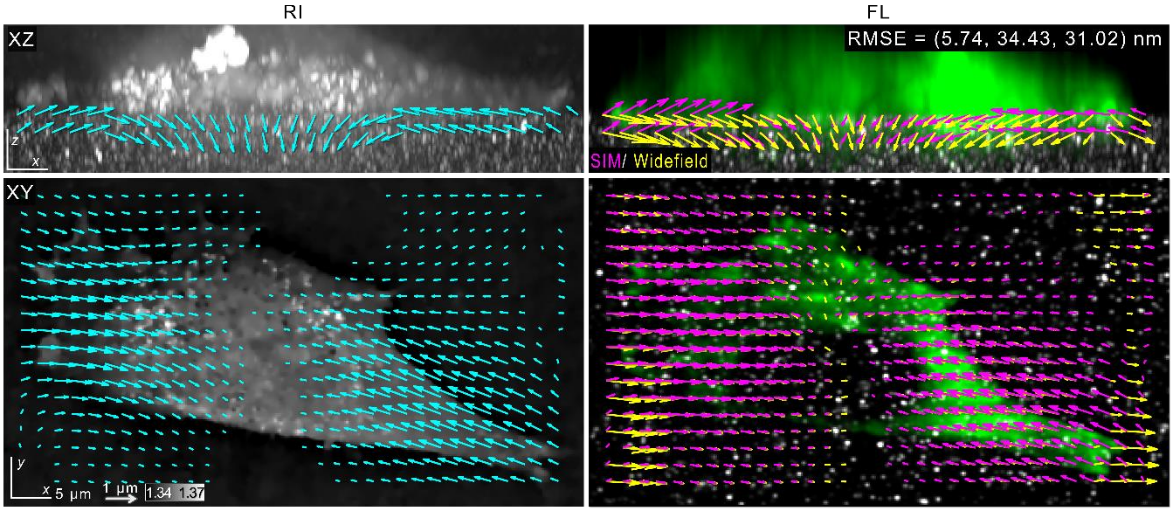

Supplementary Figure 6 | Displacement vectors computed from RI (cyan; left), 3D FL SIM (magenta; right), and 3D widefield FL (yellow; right) images.

RMSE, root mean square error between the vectors from FL-SIM and those from widefield FL along the x, y, and z directions.

Supplementary References

1. Tse, J.R. & Engler, A.J. Preparation of Hydrogel Substrates with Tunable Mechanical Properties. *Current Protocols in Cell Biology* **47**, 10.16.11-10.16.16 (2010).
