# Supplementary material for: High-resolution assessment of multidimensional cellular mechanics using label-free refractive-index traction force microscopy
Source: Commun Biol. 2024 Jan 20;7:115. doi: 10.1038/s42003-024-05788-4 (PMC10799850; doi:10.1038/s42003-024-05788-4)
Supplement: Supplementary file 3 — Description of Additional Supplementary Files [file 42003_2024_5788_MOESM3_ESM.pdf]

Description of Additional Supplementary Files

File name: Supplementary Video 1
Description: Temporal stability of RI-TFM, related to Fig. 2.

File name: Supplementary Video 2
Description: 3D rendered tomogram of a Madin-Darby canine kidney cell, related to Fig. 3.

File name: Supplementary Video 3
Description: Imaging morphology and cell traction during the rear detachment of a Madin-Darby canine kidney cell, related to Fig. 4.

File name: Supplementary Video 4
Description: Multidimensional mechanics of resting and activated CD8+ T cell on a hydrogel, related to Fig. 5.

File name: Supplementary Video 5
Description: High-speed fluctuations of protein-rich condensates and traction stress, related to Fig. 5
